# Small Intestinal Bacterial and Fungal Overgrowth: Health Implications and Management Perspectives

**DOI:** 10.3390/nu17081365

**Published:** 2025-04-17

**Authors:** Natalie Soliman, Caroline Kruithoff, Erin Marie San Valentin, Ahmed Gamal, Thomas S. McCormick, Mahmoud Ghannoum

**Affiliations:** 1 Heritage College of Osteopathic Medicine, Ohio University, Cleveland, OH 44122, USA; 2 Center for Medical Mycology and Integrated Microbiome Core, Department of Dermatology, Case Western Reserve University, Cleveland, OH 44106, USA; 3 University Hospitals St. John Medical Center, Cleveland, OH 44145, USA; 4 Department of Dermatology, University Hospitals Cleveland Medical Center, Cleveland, OH 44106, USA

**Keywords:** small intestinal bacterial overgrowth, SIBO, small intestinal fungal overgrowth, SIFO, dysbiosis, gut microbiome

## Abstract

Background/Objectives: Small Intestinal Bacterial Overgrowth (SIBO) and Small Intestinal Fungal Overgrowth (SIFO) are distinct yet often overlapping conditions characterized by an abnormal increase in microbial populations within the small intestine. SIBO results from an overgrowth of colonic bacteria, while SIFO is driven by fungal overgrowth, primarily involving *Candida* species. Both conditions present with nonspecific gastrointestinal (GI) symptoms such as bloating, abdominal pain, diarrhea, and malabsorption, making differentiation between SIBO and SIFO challenging. This review aims to elucidate the underlying mechanisms, risk factors, diagnostic challenges, and management strategies associated with SIBO and SIFO. Methods: A comprehensive review of current literature was conducted, focusing on the pathophysiology, diagnostic modalities, and therapeutic approaches for SIBO and SIFO. Results: SIBO is commonly associated with factors such as reduced gastric acid secretion, impaired gut motility, and structural abnormalities like bowel obstruction and diverticula. It is frequently diagnosed using jejunal aspirates (≥10^5^ colony forming units (CFUs)/mL) or breath tests. In contrast, SIFO is linked to prolonged antibiotic use, immunosuppression, and gut microbiome dysbiosis, with diagnosis relying on fungal cultures from small intestinal aspirates due to the absence of standardized protocols. Conclusion: The clinical overlap and frequent misdiagnosis of SIBO and SIFO highlight the need for improved diagnostic tools and a multidisciplinary approach to management. This review emphasizes the importance of understanding the mechanisms behind SIBO and SIFO, how they relate to other health outcomes, and potential management strategies to optimize patient care and therapeutic outcomes.

## 1. Introduction

The human body hosts trillions of microorganisms, collectively known as the microbiome, which play vital roles in maintaining health and influencing various physiological processes. This intricate ecosystem, comprising bacteria (bacteriome), fungi (mycobiome), viruses (virome), and archaea, is not just a collection of commensals but rather an essential extension of the human biological ecosystem [1]. While the majority of microbes reside in the gastrointestinal (GI) tract, microorganisms also inhabit the skin, the respiratory system, and the urinary/vaginal tracts, each contributing uniquely to human health.

The gut microbiota interacts with the human host through intricate biochemical pathways, contributing to vital functions such as vitamin synthesis, the breakdown of complex carbohydrates, and the regulation of immune responses. This dynamic ecosystem is uniquely shaped by genetic and environmental factors including diet, lifestyle, seasonal and hormonal changes, and the presence of specific microbes in defined niches [2]. Different anatomical sites harbor distinct microbial communities, each adapted to their unique physiological environments. For example, the acidic environment of the stomach contains fewer microbial species but often includes *Proteobacteria*. In contrast to the colon, the small intestine is characterized by lower microbial diversity and density. This is attributed to its unique environment, featuring high digestive secretions, rapid transit times (2–5 h compared to 10+ h in the colon), and protective mechanisms such as peristalsis and gastric acid [3,4].

**Figure 1:**
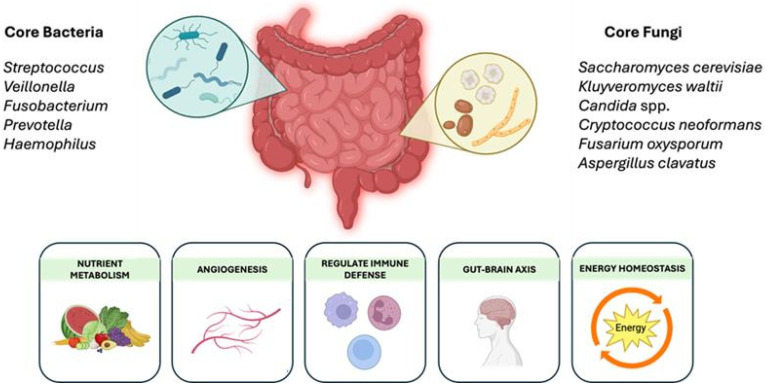
Core bacterial and fungal organisms in the small intestines play essential roles in maintaining gut health, including nutrient metabolism, angiogenesis, immune regulation, and energy homeostasis. An imbalance in these microbial communities can disrupt the relevant functions, contributing to gastrointestinal and systemic health issues. Created in BioRender (Toronto, Canada, publication license: https://BioRender.com/ry5flzn, accessed on 9 April 2025).

Under homeostatic conditions, the small intestinal microbial population is predominantly populated by *Streptococcus*, *Veillonella*, *Fusobacterium*, *Prevotella*, and *Haemophilus* bacterial genera and commensal fungi *Saccharomyces*, *Kluyveromyces*, *Candida*, *Cryptococcus*, *Fusarium*, and *Aspergillus* that contribute to essential processes, including carbohydrate fermentation, immune signaling, and maintenance of gut barrier integrity (Figure 1) [4]. The small intestine’s strategic position between the stomach and the colon makes its microbiome a critical participant in nutrient absorption and immune surveillance. Disruptions in composition can lead to microbial imbalance, known as dysbiosis, which has been implicated in numerous GI disorders. Changes in the composition and function of the small intestinal microbiota can disrupt the delicate interplay between the host immune system and the microbial community, leading to a range of adverse health outcomes.

One of the most significant consequences of small intestinal dysbiosis is the development of Small Intestinal Bacterial Overgrowth (SIBO) and Small Intestinal Fungal Overgrowth (SIFO). SIBO arises when bacterial populations in the small intestine exceed normal levels, leading to GI symptoms. Similarly, SIFO results from an overgrowth of fungal species which can cause overlapping GI symptoms (Table 1).

Understanding the small intestinal microbiome and its susceptibility to dysbiosis is critical for addressing conditions like SIBO and SIFO. This review explores the health implications of bacterial and fungal overgrowth in the small intestine, emphasizing the need for improved diagnostic and management strategies to restore microbial balance and support overall health.

## 2. Small Intestinal Bacterial Overgrowth

### 2.1. Prevalence and Pathophysiology of SIBO

In healthy individuals, less than 10^3^ organisms/mL inhabit the upper small intestine, most of which are Gram-positive bacteria. Upon the occurrence of SIBO, bacterial concentrations exceed 10^5^ to 10^6^ organisms/mL [7]. SIBO arises when there is an excess of either oral cavity bacteria (upper aerodigestive tract (UAT) SIBO) or colonic bacteria (coliform SIBO) in the small intestines. While UAT SIBO is predominantly caused by oral bacteria such as *Prevotella* and *Streptococcus viridans*, coliform SIBO is mainly caused by bacteria such as *E. coli*, *Klebsiella pneumoniae*, *Proteus mirabilis*, *Enterococcus*, or *Clostridioides* [8,9,10]. This overgrowth can interfere with digestion and nutrient absorption, leading to GI symptoms and systemic effects. SIBO patients present with a broad spectrum of symptoms, including abdominal pain, bloating, diarrhea, flatulence, and, in severe cases, malabsorption and nutrient deficiencies. The clinical presentation varies depending on both the number and the type of bacteria overgrowing in the small intestine and their metabolic byproducts [7]. For example, some bacteria may metabolize bile salts or carbohydrates and produce symptoms such as fat malabsorption [11], bloating [12], or diarrhea [13], while others may produce toxins that damage the absorptive functions of the mucosa [14].

Various factors contribute to the development of SIBO. A common misconception is that SIBO affects only a limited number of patients, such as those with an anatomic abnormality of the upper GI tract or those with motility disorders [7]. Gut microbiome dysbiosis can result from both modifiable and non-modifiable factors. Modifiable factors include lifestyle habits such as diet, exercise, smoking, stress, and sleep. For example, diets high in simple carbohydrates and sugar or diets low in fiber and antioxidants, as well as vitamin deficiencies, can promote bacterial or fungal overgrowth [15]. Non-modifiable factors include genetic predisposition, age, gender, and immunocompromised status. Certain medications may also contribute to dysbiosis, including antibiotics [16], narcotics [17], corticosteroids [18], non-steroidal anti-inflammatory drugs (NSAIDs) [19], and proton pump inhibitors (PPIs) [20,21].

SIBO is more prevalent in individuals with underlying health conditions, particularly those with chronic diseases and conditions such as liver disease [22], obesity [23], irritable bowel syndrome (IBS) [17,24,25], inflammatory bowel disease (IBD) [26], gastric bypass surgery [27], dyspepsia [28], lactose intolerance [29], *Helicobacter pylori* (*H. pylori*) infection [30], and diabetes mellitus. Increased prevalence is also observed among individuals with a history of antibiotic use [31]. For example, recent studies have re-examined the prevalence of SIBO in IBD patients. It has been reported in up to 62% of people with this syndrome, including those with ulcerative colitis and Crohn’s disease (CD), and is associated with symptoms indicating active disease [32,33,34]. Age-related declines in gastric acid production and intestinal motility further increase susceptibility in older adults, who report SIBO prevalence rates of 14.5% to 15.6% based on hydrogen breath testing [17,35,36]. In addition, certain conditions affecting gut motility and immune function, such as systemic lupus erythematosus [37], systemic scleroderma [38], Marfan syndrome [39], and various vasculitides [40], may increase the risk of SIBO [39,40].

Gut microbiome composition varies across populations due to genetic factors, diet, and environmental influences. Geographic variations in SIBO prevalence have been reported among IBS patients, with rates of 10% in Canada, 23% in Europe, 14% in India, 37% in Iran and Southeast Asia, and 55% in the United States [41]. However, conflicting findings in different geographic regions may be related to the differences in diagnostic criteria and methodologies used [13,26,42]. Some studies suggest females may be at higher risk of developing SIBO [24,43], while others indicate no association between SIBO and gender or race [43].

Older adults may be more susceptible to SIBO due to age-related changes, including reduced gastric acid levels, slowed gut motility, and the increased use of medications such as NSAIDs and PPIs [44]. Older age is also considered a significant risk factor for SIBO in Crohn’s disease patients [44]. In a study of 294 non-hospitalized older adults with 34 younger adults (average age 33.6 years) as controls, SIBO prevalence, measured by the glucose breath test, was 5.9% in the younger group and 15.6% in the older group [35]. Similarly, a study in the United Kingdom found that 14.5% of healthy elderly volunteers tested positive for SIBO using the glucose breath test [36]. These studies support the concept that SIBO prevalence increases with age.

### 2.2. Methods Used to Diagnose SIBO

The presence of SIBO may be suggested clinically by symptoms of abdominal pain, bloating, and chronic diarrhea; however, it is necessary to confirm the diagnosis with objective testing. The current gold standards for the diagnosis of SIBO are jejunal aspiration and quantitative culture, which directly measure bacterial counts within the small intestine [45]. A positive diagnosis for SIBO involves detecting ≥10^5^ bacterial CFUs/mL of proximal jejunal aspiration, compared to a normal value of ≤10^3^ CFUs/mL [46]. Although this procedure provides a definitive diagnosis, it is invasive, costly, time-consuming, and infrequently accessible given the current primary care medical infrastructure. This method also lacks standardized techniques of sample collection and typically risks sample contamination. In place of jejunal aspiration, hydrogen breath testing, a simple, non-invasive, and inexpensive measure, has more recently been implemented to diagnose suspected SIBO. This technique measures the concentration of gases such as hydrogen, methane, or hydrogen sulfide in the exhaled air following the oral administration of lactulose or glucose [47]. While the sensitivity of the hydrogen breath test varies depending on the substrate used, the additional measurement of methane can increase the sensitivity of the test [45]. However, the sensitivity and specificity of the breath test method are still limited [47,48]. In addition, the equipment necessary to detect these gases may be cost-prohibitive, making widespread adoption difficult to implement.

Using the above methods of gas-based detection, SIBO can be further classified into three main subtypes. First, hydrogen-dominant SIBO is characterized by excessive hydrogen production by bacteria such as *E. coli* and *Klebsiella* [49]. This type of SIBO is often associated with diarrhea as a predominant symptom. On the other hand, the overgrowth of methane-producing archaea, particularly *Methanobrevibacter smithii*, is a key feature in the methane-dominant subtype of SIBO. Methane is known to slow intestinal transit, leading to constipation as the predominant symptom [50]. The third subtype, hydrogen sulfide SIBO, is less commonly diagnosed. In this subtype, hydrogen sulfide-producing bacteria such as *Vibrio* and *Desulfovibrio* contribute to the main symptomologies, including diarrhea, bloating, and in some cases, more severe GI symptoms [51,52].

### 2.3. Mechanisms Linking SIBO to Diseases

SIBO occurs when the usual mechanisms that regulate bacteria levels in the gut—such as intestinal motility [53], gastric acid secretion [54], and immune surveillance [55]—are disrupted, leading to an excessive population of bacteria in the small intestine [7]. Several key underlying factors contribute to this disruption. One significant factor is the formation of biofilms, which are communities of bacteria, encased in a self-produced extracellular matrix, that adhere to the intestinal mucosa. Biofilms allow pathogenic bacteria such as *E. coli*, *Klebsiella pneumoniae*, and *Enterococcus* spp. to persist despite antimicrobial treatments, thus contributing to the chronic and recurrent nature of SIBO. Biofilms have been demonstrated to decrease the effectiveness of using antibiotics to treat bacterial infections, as well as the diminution of host immune defense, making standard treatment less effective and increasing the likelihood of sustained bacterial overgrowth [56,57]

Another key regulatory mechanism is gastric acid production, which typically controls the growth of bacteria, keeping bacterial levels low in the upper small intestine. Hypochlorhydria, or reduced acid production, thus increases the risk of SIBO and can occur following *H. pylori* infection. SIBO may also arise following the consumption of certain medications such as PPIs and histamine type 2 receptor blockers (H2RAs), which reduce stomach acid secretion [58,59,60]. In fact, investigators reported that 53% of adult patients taking omeprazole, a commonly used PPI, had SIBO [54]. Another contributing factor is that the incidence of SIBO also appears to increase with age [61,62].

**Figure 2:**
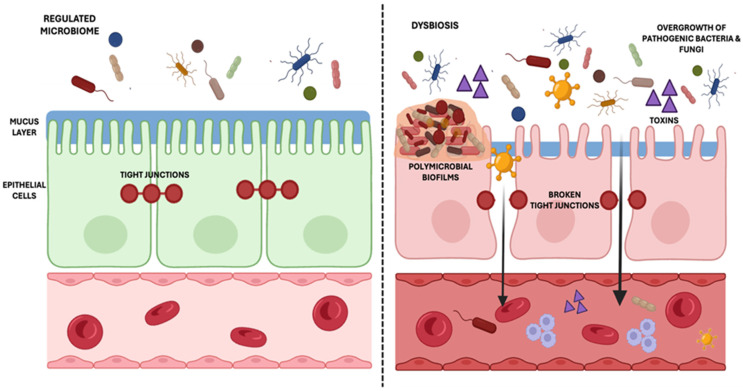
A normal, well-balanced microbiome supports healthy, tight junctions in the intestinal lining, maintaining its selective permeability. However, dysbiosis and pathogenic biofilm formation can compromise these tight junctions, allowing harmful substances to enter the bloodstream and trigger systemic inflammation. In SIBO, bacterial metabolites such as lipopolysaccharides (LPSs), hydrogen sulfide (H_2_S), and methane disrupt epithelial integrity, contributing to increased permeability. In SIFO, fungal enzymes such as phospholipases and aspartic proteinases further exacerbate gut barrier dysfunction. This increased permeability, often referred to as “leaky gut”, is linked to chronic diseases, autoimmune disorders, and other inflammatory health issues associated with poor diet and gut microbiota imbalances. Created in BioRender (Toronto, Canada, publication license: https://BioRender.com/nlrx413, accessed on 9 April 2025).

The dysbiosis brought about by SIBO is also known to increase intestinal permeability (Figure 2), which facilitates the translocation of bacterial endotoxins and triggers systemic inflammation. This mechanism is particularly relevant in a range of chronic diseases, including obesity and diabetes. The role of lipopolysaccharides (LPSs) and Toll-like receptor 4 (TLR-4) signaling in promoting pro-inflammatory cytokine release and contributing to insulin resistance and metabolic dysfunction may also be relevant in the context of SIBO [63,64]. The overgrowth of bacteria in SIBO could lead to increased LPS production [65], activating TLR-4 signaling and triggering the release of pro-inflammatory cytokines such as TNF-α, IL-6, and IL-1β [66]. Studies have shown that obese individuals are more likely to have SIBO, where dietary patterns further contribute to SIBO diagnosis [67,68]. In this regard, obese individuals with SIBO are reported to consume more carbohydrates and refined sugars and have a lower intake of total and insoluble fibers compared to those without SIBO. Furthermore, in obese children, the prevalence of SIBO was significantly higher compared to non-obese counterparts (37.6% vs. 3.3%; OR = 17.5) [69].

Emerging evidence also suggests that there is a potential link between Glucagon-Like Peptide-1 (GLP-1) agonists and SIBO. GLP-1 agonists, including semaglutide, liraglutide, and dulaglutide, are medications used to treat diabetes and obesity. They have increased in popularity, particularly for weight loss. These medications delay gastric emptying and reduce gut motility while stimulating insulin release [70]. Interestingly, GLP-1 agonists may alter the gut microbiome, potentially increasing the risk of bacterial and fungal overgrowth. For instance, research in murine models has shown that liraglutide increases *E. coli* levels in the gut by activating sympathetic activity and norepinephrine release and can also lead to bacterial translocation and changes in intestinal tight junction genes in colitis models [71]. Furthermore, a case study documented worsened lactose intolerance and newly developed gluten intolerance in a patient taking oral semaglutide, with symptoms persisting even after discontinuing the medication and a subsequent diagnosis of SIBO [72]. Additionally, a retrospective case–control study found an association between the use of weight loss medications, specifically GLP-1 agonists, and both intestinal methanogen overgrowth and hydrogen SIBO [73]. While SIBO has previously been linked to diabetes and obesity, this evidence suggests the existence of a possible independent association between hydrogen SIBO and the use of GLP-1 agonists.

A similar mechanism of microbial dysbiosis can be hypothesized for the association of SIBO in colorectal cancer, where gut microbiome dysbiosis is postulated to influence adenoma–carcinoma progression [74]. Risk factors such as obesity, smoking, alcohol consumption, and high-fat diets contribute to this imbalance. Specific bacteria such as *Fusobacterium nucleatum*, *E. coli*, *Enterococcus faecalis*, *Streptococcus gallolyticus*, and enterotoxigenic *Bacteroides fragilis* have been found to be associated with the onset and progression of colorectal cancer [75,76]. Particular attention has been paid to *Streptococcus gallolyticus*, as evidence has shown that it contains tumor-promoting capabilities within the colon and is associated with key cytokines and transcriptional factors, such as IL-1, IFN-γ, IL-8, and NF-kB [77,78], involved in colorectal tumorigenesis. These findings suggest that SIBO and the resulting dysbiosis may contribute to carcinogenesis by fostering a pro-inflammatory and tumorigenic microenvironment. Further research is needed to support the concept that SIBO may be linked to cancer.

The liver and gut maintain close two-way communication through the portal circulation system and biliary tree. While the liver receives gut-derived substances (e.g., bacterial metabolites and food antigens) and in turn drives molecules (cytokines, bile acids, and hormones) to regulate the gut microbiome, disruptions such as alcohol consumption, inflammation, and dysbiosis can increase intestinal permeability [79]. This exposes the liver to more pro-inflammatory molecules, activating the immune system and causing hepatic and systemic inflammation. Consequently, SIBO is also commonly observed in patients with liver disorders such as hepatocellular carcinoma and minimal hepatic encephalopathy [80].

Several studies have also reported the occurrence of SIBO in dermatologic disorders such as acne, rosacea, psoriasis, and atopic dermatitis, showing evidence of a bidirectional relationship between gut health and skin (gut–skin axis) [81,82]. This relationship is exemplified in systemic sclerosis (SSc), also known as scleroderma, a chronic autoimmune disorder characterized by the hardening and thickening of skin and connective tissues. After the skin, the GI tract represents the second most affected site in SSc patients, with impacts often leading to intestinal dysmotility and microbial growth. A study involving 55 SSc patients demonstrated that 56% of these patients also had SIBO [83]. Treatment with rifaximin resulted in the significant (*p* < 0.05) eradication of intestinal symptoms in 73.3% of these patients, suggesting the existence of a strong association between SIBO and GI symptoms experienced in cases of SSc.

Similarly, rosacea has been associated with various GI diseases including IBD, celiac disease, IBS, gastroesophageal reflux disease (GERD), *H. pylori* infection, and SIBO. A prospective study found SIBO in 46% of patients (*n* = 113) with rosacea, and its prevalence was significantly higher than in healthy controls (*p* < 0.001) [84]. Most of the SIBO-positive patients with rosacea presented with papulopustular-type rosacea. At the end of a 10-day treatment course with rifaximin, almost complete regression of cutaneous lesions was also observed, likely due to the reduction in systemic inflammation mediated by TNF-α, which is induced by dysbiosis in SIBO. Interestingly, rosacea symptoms did not improve with rifaximin treatment in patients without SIBO, further supporting the role of circulating TNF-α in this relationship. A case report by Weinstock [85] described three patients with Crohn’s disease who also had rosacea, all of whom experienced both GI and skin symptom improvement after 10-day treatment with rifaximin. While SIBO was not formally tested, the resolution of symptoms suggests that the effect of the antibiotic treatment may be mediated through the modulation of the gut flora, supporting the existence of a link between SIBO, dysbiosis, and immune regulation in rosacea.

While the published studies are contradictory, the same mechanism is hypothesized to be present in the relationship between psoriasis and SIBO. Psoriasis is another chronic inflammatory skin disease characterized by erythematous and scaly plaques, commonly affecting the elbows, scalp, trunk, and knees [86]. SIBO treatment has been associated with the amelioration of cutaneous lesions [87,88]. In other words, treating and eliminating SIBO can lead to better skin health and a reduction in skin disorders. Additionally, there are accounts of SIBO treatment’s effectiveness when it comes to oral psoriasis manifestations (geographic and fissured tongue) [89]. Essentially, eradicating SIBO may have a positive impact on specific oral and dermatologic symptoms associated with psoriasis.

## 3. Small Intestinal Fungal Overgrowth

### 3.1. Prevalence and Pathophysiology of SIFO

SIFO is a form of dysbiosis characterized by the presence of excess fungi in the small intestine, typically defined as more than 1000 fungal CFUs/mL of small intestinal aspirate [90]. Fungal infection, particularly candidiasis, is known to cause GI symptoms in patients with underlying diseases such as cancer and diabetes mellitus, and in those receiving steroids or antibiotics [91]. However, SIFO can also affect healthy and immunocompetent individuals, causing GI complaints. The incidence and prevalence of SIFO are not as well-documented as those of SIBO. However, factors that contribute to dysbiosis and increase the risk of SIBO may also increase the risk of SIFO.

The symptoms of SIFO often overlap with those of other GI disorders, making diagnosis challenging. Patients with SIFO, especially those with comorbidities such as diabetes or those receiving antibiotic or chemotherapy treatments, may present with watery diarrhea, mucus in the stool, urgency and bloating, and severe abdominal pain [65,92]. Additionally, immunocompromised individuals with fungal overgrowth have reported experiencing chest pains, belching, indigestion, and gas [90].

Populations at higher risk for SIFO primarily include immunocompromised individuals, such as those with human immunodeficiency virus (HIV), cancer, diabetes, or organ transplants [65]. Similarly to SIBO, PPI use and prolonged antibiotic use may impair small intestine peristalsis, increasing the risk of SIFO [93]. Dysmotility is an independent risk factor for both SIBO and SIFO [93]. These conditions may occur concurrently, with about 34% of SIBO patients also being eventually diagnosed with SIFO [93]. The co-occurrence of these conditions is also noted in patients with a history of colectomy or chronic unexplained GI symptoms [90]. Further research is needed to explore the epidemiology of SIFO and provide more insight into its predisposing risk factors.

### 3.2. Methods Used to Diagnose SIFO

Although similar limitations exist in diagnosing SIFO compared to SIBO diagnosing SIFO is particularly challenging due to the lack of standardized testing methods. The current gold standard for the diagnosis of SIFO involves culturing aspirated fluid from the distal duodenum or jejunum, but this method is invasive and not widely available. A commonly used arbitrary cutoff for diagnosing SIFO is a fungal burden ≥ 10^3^ CFUs/mL [65,91]. Fungal colonization does not always indicate pathological overgrowth and may be part of the normal microbiome, limiting the specificity of aspiration testing. Additionally, unlike SIBO, breath testing is not a reliable diagnostic tool for fungal overgrowth as fungi do not produce measurable hydrogen or methane gases during fermentation and, as mentioned above, the test is designed to detect bacterial, not fungal, metabolism in the small intestine [45]. Since SIBO and SIFO may coexist and present with similar symptoms, distinguishing whether symptoms are due to fungal overgrowth alone is difficult. If a patient exhibits symptoms suggestive of SIBO but does not respond to antibiotic therapy, SIFO should be considered [47]. However, the absence of universally accepted diagnostic guidelines has led to inconsistent clinical approaches to managing SIFO. Due to these significant diagnostic challenges, SIFO remains a controversial and often underdiagnosed condition. Further research is needed to develop less invasive diagnostic methods and define clear clinical guidelines.

### 3.3. Mechanisms Linking SIFO to Diseases

While research exploring the link between SIFO and diseases is less extensive than that for SIBO, the following mechanisms are proposed to illustrate how SIFO may contribute to various health conditions. *Candida* species are the key players identified in SIFO. While they primarily colonize human mucosal surfaces and skin, the presence of *Candida* in the gut is well documented [94,95,96,97] and is hypothesized to be regulated by the host immune system as well as microbial antagonism under homeostatic conditions [98]. For example, beneficial bacteria such as *Lactobacillus* spp., can inhibit the growth of *Candida* by reducing growth, cell adhesion, and filamentation [99]. Thus, it is expected that the depletion of *Lactobacillus* as a result of antibiotic use may facilitate the overgrowth of *Candida*. While *Candida albicans* is the most commonly associated species in SIFO, other species within the *Candida* genus can be involved as well, including *C. glabrata*, *C. tropicalis*, *C. krusei*, *C. famata*, and *C. parapsilosis.* Certain patient populations may exhibit species-specific dysbiosis such as those individuals with Crohn’s disease, wherein *C. tropicalis* has been identified as a dominant fungal species contributing to gut inflammation [97], although it often may be misidentified as a *C. albicans* [100].

**Figure 3:**
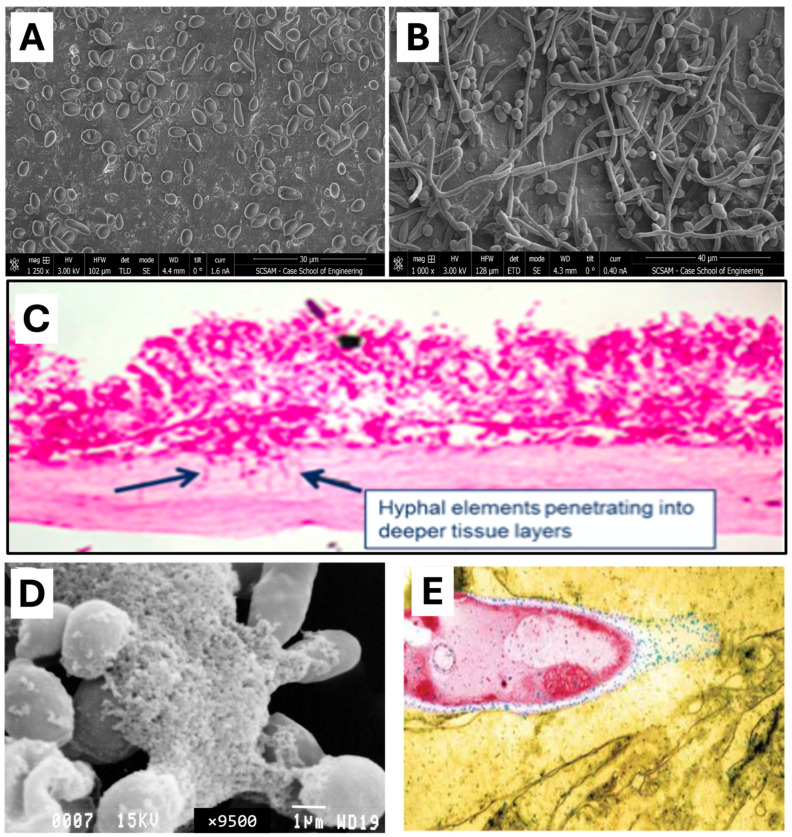
Illustration of Candida virulence factors. (**A**) Scanning Electron Microscopy image of *Candida* showing its phenotypic plasticity either as oval-shaped budding cells or (**B**) thread-like structures called hyphae. (**C**) Microscopy image showing invasive growth of fungal hyphae, indicative of epithelial barrier damage in mouse model (image from Ghannoum [101]). (**D**) Biofilm formation of *Candida* demonstrating creation of complex structure that could invade and break down gastrointestinal (GI) lining (image from Ghannoum [102]). (**E**) Phospholipase production (blue dots) during *Candida albicans* invasion aids in fungi invasion of GI cell: magnification × 10,000 (image from Ghannoum [103]).

Several factors can lead to fungal overgrowth and cause damage to the mucosal barrier, including inherent *Candida* virulence factors that enhance their pathogenicity. First, *Candida* is known to exhibit phenotypic plasticity, enabling it to transition between distinct morphological forms. This capacity for morphological switching, primarily between yeast (Figure 3A) and hyphal forms (Figure 3B), is central to candidal pathogenicity. The yeast form facilitates dissemination within the host, while the hyphal form promotes tissue invasion and damage (Figure 3C). This dimorphic transition is highly responsive to environmental cues, including pH, temperature, and nutrient availability, allowing *Candida* to adapt to diverse host niches.

A second virulence factor of *Candida* is its ability to form biofilms (Figure 3D). Biofilms are communities of microbial cells that adhere to surfaces, forming a matrix that makes them resistant to antimicrobials as well as host defenses [104,105]. In this regard, the relationship between fungal and bacterial communities in the gut also plays an important role in the pathogenesis of SIFO. A balanced gut microbiome can prevent fungal overgrowth through competitive inhibition and microbial interactions. Beneficial bacteria such as *Lactobacillus* contribute to this balance by producing hydrogen peroxide, which has antifungal properties that suppress *Candida* growth [106]. Similarly, the beneficial yeast *Saccharomyces boulardii* inhibits *Candida* growth by preventing its adhesion to the intestinal mucosa and disrupting its biofilm formation [103,107]. On the other hand, certain bacterial species can promote fungal persistence, leading to a shift in the microbial equilibrium. For example, emerging research has highlighted the role of fungal dysbiosis in CD, particularly in the interactions between *C. tropicalis* or *C. albicans*, *E. coli*, and *Serratia marcescens*. These microorganisms have been shown to form polymicrobial biofilms in the intestinal mucosa of CD patients, contributing to inflammation and disease progression. *C. albicans* enhances the persistence of *E. coli*, while *S. marcescens* promotes the degradation of intestinal barriers, exacerbating inflammation. The formation of biofilms may then promote fungal overgrowth, which impedes nutrient absorption and damages the intestinal epithelia resulting in leaky gut, further complicating dysbiosis in CD and increasing the likelihood of SIFO [104,108].

Lastly, *Candida* species also release enzymes, such as phospholipases and aspartic proteinases, that can disrupt epithelial integrity and contribute to systemic inflammation [109] (Figure 3E). These enzymes may impair nutrient absorption, trigger oxidative stress, and induce immune activation. The direct connection between IBS and SIFO calls for more studies; it is hypothesized that yeast antigens, particularly from *Candida*, could potentially contribute to IBS symptoms [95]. *Candia* produces alcohol and glycoproteins that stimulate mast cells to release inflammatory substances such as histamine and prostaglandin [110]. Additionally, *Candida* produces proteases that degrade secretory IgA, a key antibody protecting the intestinal lining [111]. This degradation could lead to inflammation and polyclonal B-cell response, potentially explaining why some IBS patients experience symptoms linked to *Candida* infection. Similarly, SIFO may be implicated in insulin resistance, obesity, and diabetes through the same pathway(s). Studies suggest that *Candida* overgrowth activates Th17-mediated inflammation [112], further contributing to insulin resistance and the metabolic challenges observed in obesity and diabetes [113,114].

Fungal dysbiosis in the gut has also been implicated in the development of colorectal cancer. Studies reported the presence of *Candida* species, particularly *C. albicans*, in colorectal tumors, suggesting the existence of a potential link between fungal overgrowth and carcinogenesis through inflammation, epithelial damage, and immune dysregulation [115,116,117]. Additionally, *Candida* species also produce potentially carcinogenic byproducts, such as acetaldehyde, and increase the production of hydrolases, which may trigger chronic inflammatory responses in host tissues [118].

SIFO can also disrupt normal digestive processes. This may lead to the malabsorption of nutrients, resulting in malnutrition, as the body is deprived of the essential nutrients necessary for maintaining health. Symptoms such as chronic diarrhea further exacerbate nutrient loss and contribute to weight loss, as highlighted in a case-study of a 48-year-old female with Sjögren’s syndrome and cervical cancer [100]. Diagnostic procedures revealed an overgrowth of *C. tropicalis* in the patient’s small bowel. Following antifungal treatment and nutritional support, the patient showed marked improvement, underscoring the critical role of addressing fungal overgrowth in such clinical diagnoses.

## 4. Treatment and Management Strategies for SIBO and SIFO

Traditionally, SIBO and SIFO are mainly treated with antibacterial and antifungal agents, respectively. For example, antibiotics such as rifaximin can be prescribed for 10–14 days, with approximately 60–70% efficacy [119,120]. A significant observation from studies is that rifaximin behaves as a eubiotic in the GI tract, suggesting that it supports the intestinal microbiota by enhancing beneficial bacteria like *Lactobacillus* and *Bifidobacterium.* Furthermore, rifaximin also reduces inflammation, strengthens intestinal barrier function, and helps to prevent bacterial translocation [121]. SIFO, like other fungal infections, can be treated with antifungals such as azoles (e.g., fluconazole) [92] and polyenes (e.g., amphotericin B and nystatin) [91,92].

However, there is also evidence that antibiotic use results in low-to-moderate efficacy for the treatment of SIBO. Specifically, a study of 80 SIBO patients showed that the recurrence of SIBO symptoms was observed in 12.6% of the subjects after 3 months, 27.5% after 6 months, and 43.7% after 9 months [122] of treatment with antibiotics. More recent guidelines from the American College of Gastroenterology (ACG) [123] on SIBO recommend using the antibiotic trimethoprim-sulfamethoxazole in combination with metronidazole, a practice which has shown a 95% response rate in a pediatric SIBO cohort (*n* = 20) [124]. However, it is also important to highlight that repeating antibiotic treatment may in turn increase the risk of antibiotic resistance, diarrhea, *Clostridioides* infection, intolerance, and gut microbiome imbalance [125].

In cases where SIBO and SIFO may be recurrent or relapsing, the use of alternative strategies and combination therapies has become the preferred approach [126,127]. These approaches, discussed in further detail below, include combining antibiotics or antifungals with probiotics and dietary and lifestyle modifications, which can help restore microbial balance, support intestinal health, and reduce relapse risk.

The fecal microbiota transplantation (FMT) for SIBO patients, for example, is an emerging strategy and has been previously explored in a randomized, placebo-controlled trial. It was demonstrated that participants given FMT capsules (*n* = 28) displayed significantly (*p* < 0.05) improved GI symptoms (measured using the Gastrointestinal Symptom Rating Scale) at one, three, and six months post-treatment compared to the participants in the placebo group [128]. This study noted, however, that findings should be verified in a larger clinical trial.

Although surgical interventions are not typically used in the management of SIBO and SIFO, they may be indicated in SIBO patients displaying the prolonged failure of initial conservative measures [129], or in those with anatomic abnormalities and recurrent SIBO [130]. For example, surgical treatment may be beneficial in cases of SIBO secondary to GI complications like small bowel diverticulosis, fistulas, or strictures [7]. To date, we are unaware of any documented cases of surgical intervention specifically for the management or treatment of SIFO.

A common management strategy for both SIBO and SIFO is dietary modulation. A diet that restricts fermentable oligosaccharides, disaccharides, monosaccharides, and polyols is known as a low-FODMAP diet and is used to treat IBS, limiting the microbial energy sources needed for growth. This restriction reduces bacterial fermentation, as indicated by the reduced levels of hydrogen observed in breath tests [131,132]. However, it is important to note that the complete elimination of FODMAPs from the diet of SIBO patients is not recommended to exceed six weeks [123,133]. If this approach proves ineffective, it should not be reintroduced as a subsequent treatment.

The diet plan outlined in *Total Gut Balance* [5] (also referred to as the mycobiome diet) is another clinically studied diet plan that limits the growth of pathogenic bacteria and fungi, while fostering and nourishing beneficial bacteria and fungi in the gut. A 28-day study was conducted on the mycobiome diet, with healthy volunteers aged 30–70 years, to assess its impact on the gut’s microbiome composition and overall health [6]. Participants followed a structured dietary regimen while tracking microbiome changes, digestive symptoms, and other health measures. The results demonstrated a significant reduction in the abundance of *Candida* genus, with a 72.4% overall decrease and the complete elimination of *C. tropicalis* after four weeks. A notable increase in beneficial fungal species, such as *Galactomyces geotrichum* and *Pichia kluyveri*, contributed to the promotion of microbial balance. Additionally, there was a marked decrease in pro-inflammatory *Proteobacteria* phylum and pathogenic bacteria, including *E. coli* and *Clostridium*, alongside increased levels of beneficial bacteria such as *Faecalibacterium prausnitzii*, *Bifidobacterium adolescentis*, and *Lactobacillus*. Subjective self-reported health improvements included reduced GI symptoms, weight loss, increased energy, and improved sleep, particularly among participants who previously reported these issues.

Dietary supplements may also help to alleviate SIBO and SIFO symptoms. Ginger has been shown to improve gut motility and accelerate gastric emptying, reduce pressure on the lower esophageal sphincter, and prevent bloating and flatulence [53]. Licorice contains an anti-inflammatory flavonoid, glycyrrhizin, and has an antimicrobial effect against *E. coli*. There are many other food products/ingredients reported to possess antimicrobial effects, including garlic/allicin, turmeric/curcumin, oregano/carvacrol, thyme/thymol, rosemary, cinnamon, and onion. Supplementation with probiotics, which are live microorganisms that can provide health benefits to the host [134], is particularly valuable because they can influence the composition of intestinal microbiota and shield the gut from pathogen colonization. Because of these beneficial properties, there have been attempts to supplement SIBO treatment with probiotic strains that have demonstrated clinical effects [135,136,137]. Although most studies have been small and focused on different outcomes, a 2017 meta-analysis of probiotic therapy for SIBO found that probiotics reduce hydrogen levels detected in breath tests (odds ratio (OR) = 1.61, 95% confidence interval (CI) = 1.19–2.17), indicating a potential decrease in fermentative bacterial overgrowth [138].

It is crucial to note that, as with other conditions, the effectiveness of probiotic therapy in SIBO and SIFO varies significantly with strain/s used, and not all probiotics are equally effective. For example, a 2018 study found that administering probiotics to SIBO patients led to worsened bloating, flatulence, metabolic lactic acidosis, and “brain fog.” These symptoms improved after discontinuing the probiotics and starting antibiotic treatment [12].

A probiotic blend, comprising *Bifidobacterium breve*, *Saccharomyces boulardii*, *Lactobacillus acidophilus*, *Lactobacillus rhamnosus*, and the digestive enzyme amylase (Mycohsa™, BIOHM Technologies, Cleveland, OH, USA), was developed specifically to rebalance the bacterial and fungal gut microbiome, reduce inflammation, and support a healthy gut population [139]. The gut bacterial and fungal communities of 49 healthy participants were evaluated using stool samples collected at baseline and after 4 weeks of daily consumption. This led to a significant reduction in the abundance of the *Candida* genus (*p* < 0.013), with a decreasing trend in *C. albicans*, although it was not statistically significant. For bacteria, a reduction was noted in *Firmicutes* at the phylum level, approaching levels reported for control individuals observed in the Human Microbiome Project [140,141]. These preliminary findings suggested that the probiotic could rebalance the gut microbiome in healthy individuals, warranting further trials to explore its potential benefits in individuals with GI symptoms, particularly those with SIBO and SIFO.

Moreover, in the context of SIBO and SIFO, metabolic byproducts of probiotics (“postbiotics”) are gaining attention for their potential to modulate the gut environment without introducing live organisms. This is particularly relevant as SIBO and SIFO are characterized by an imbalance in the small intestine’s microbial ecosystem, where introducing more live organisms may not always be beneficial [142]. Future research should also investigate the potential role of postbiotics in managing these conditions, given their capacity for the targeted modulation of the gut environment without the risks associated with live probiotics in sensitive individuals.

Other lifestyle modifications may also play a crucial role in managing SIBO and SIFO, reducing stress and increasing sleep and physical activity, effects which are also likely to impact gut health and microbial balance. Regular exercise has been shown to promote gut microbial diversity and enhance gut barrier function. Studies indicate that higher fitness levels correlate with increased levels of beneficial bacteria, such as *Faecalibacterium prausnitzii* [143] and butyrate-producing species (*Clostridiales*, *Roseburia*, *Lachnospiraceae*, and *Erysipelotrichaceae*) [144], which support gut health and reduce inflammation. Additionally, exercise has led to higher levels of *Akkermansia muciniphila*, a bacteria reported to be associated with lower BMI and metabolic health [143]. Beyond exercise, minor lifestyle changes such as stress management (e.g., practicing meditation and yoga) and obtaining adequate sleep may further support gut balance and alleviate symptoms of SIBO and SIFO.

## 5. Future Directions in SIBO and SIFO Research

Despite growing recognition of SIBO and SIFO as contributors to GI dysfunction, significant gaps remain in our understanding of the pathophysiology, diagnosis, and treatment of these disorders. One major limitation is the lack of standardized diagnostic criteria and reliable testing methods. While glucose and lactulose breath tests are commonly used for SIBO, their sensitivity and specificity vary, often leading to false positive or negative outcomes. Similarly, SIFO diagnosis relies on duodenal aspirate cultures, which are invasive, not widely available, and may not accurately reflect fungal overgrowth throughout the small intestine.

Another gap in the current knowledge is the role of biofilm formation in SIBO and SIFO persistence. Biofilms may explain why many patients experience recurrent symptoms despite antibiotic or antifungal treatment. However, research on biofilms in the small intestine is still limited. Additionally, exploring the cross-kingdom interactions between bacterial and fungal communities within these biofilms could provide new insights into treatment-resistant cases and help to develop targeted therapeutic interventions.

Beyond the challenges in diagnosis and treatment, there is increasing recognition that SIBO and SIFO are not merely consequences of different pathological conditions, but may also actively contribute to disease progression, creating a bidirectional relationship. Conditions such as GI motility disorders, immunodeficiencies, and gastric acid suppression can predispose individuals to SIBO and SIFO; yet, once, established, these microbial imbalances can further exacerbate systemic inflammation, intestinal permeability, and nutrient malabsorption. This cyclical interaction suggests that gut dysbiosis may result from as well as drive chronic diseases, highlighting the need for therapeutic approaches that target both the underlying condition and microbial growth.

Given that the current data on SBO and SIFO primarily come from small-scale studies, it is important to conduct larger studies, utilizing bioinformatics and artificial intelligence (AI) approaches to gain deeper insights into these conditions. Beyond microbiome rebalancing, further research is needed to explore the complex relationship between gut dysbiosis and systemic health. SIBO and SIFO have been linked to a range of conditions, including metabolic disorders, autoimmune diseases, and neurological conditions. In this context, leveraging AI and bioinformatics tools can enhance the identification of microbial biomarkers, improve prognostic and therapeutic predictions, and support the development of personalized treatment strategies for SIBO and SIFO.

## 6. Conclusions

SIBO and SIFO represent significant yet often overlooked contributors to a range of GI and systemic diseases. Their associations with a broad variety of diseases highlight the critical role of microbial dysbiosis in disease and pathogenesis. Emerging evidence suggests that SIBO and SIFO may contribute to inflammation and immune dysregulation, leading to GI symptoms and potentially influencing the development of skin disorders, neurological conditions, metabolic issues, and other systemic diseases. While the current research provides valuable insights, gaps remain in fully understanding SIBO and SIFO, as well elucidating the mechanisms by which they contribute to diseases. Future studies should focus on improving diagnostic methods, exploring targeted therapies, and integrating gut microbiome modulation into disease management strategies.

## Figures and Tables

**Table 1:** Comparative features of small intestinal bacterial overgrowth and small intestinal fungal overgrowth.

Features	Small Intestinal Bacterial Overgrowth (SIBO)	Small Intestinal Fungal Overgrowth (SIFO)
Etiology	Overgrowth of bacteria (e.g., *E. coli*, *Klebsiella*, *Streptococcus*)	Overgrowth of fungi (mainly *Candida* species)
Risk Factors	Hypochlorhydria (e.g., PPI use) Impaired motility Anatomical abnormalities Aging	Antifungal or steroid use immunosuppression Diabetes, cancer, or malnutrition
Prevalence	Higher in patients with IBS, IBD, liver disease, obesity, elderly; exact prevalence unclear	Under-recognized; up to 34% of SIBO patients may have coexisting SIFO
Pathogenesis	Bacterial overgrowth leads to nutrient malabsorption, bile salt de-conjugation, gas production, leaky gut	Fungal overgrowth disrupts epithelial integrity, forms biofilms, and causes virulence enzymes
Symptoms	Bloating, diarrhea, constipation (depending on gas type), flatulence, malabsorption	Abdominal pain, bloating, diarrhea, indigestion, gas; often mimics other GI disorders
Diagnostic Methods	Gold standard: jejunal aspirate (>10^5^ CFUs/mL) Breath tests (hydrogen, methane)	Gold standard: fungal culture from small intestinal aspirate Clinical suspicion
Antimicrobial Therapy	Rifaximin, TMP-SMX + Metronidazole	Fluconazole, Nystatin, Amphotericin B
Dietary Approaches	Low FODMAP diet (≤6 weeks), mycobiome diet [5,6]	Mycobiome diet
Probiotics	Mixed results; strain-specific	*Saccharomyces boulardii* may help
Herbal/Botanical Agents	Ginger, garlic (allicin), turmeric, oregano, etc.	Same as SIBO
Lifestyle Modifications	Stress reduction, sleep, exercise	Same as SIBO

Abbreviations: PPI: proton pump inhibitor; IBS: irritable bowel syndrome, IBD: irritable bowel disease; GI: gastrointestinal; TMP: trimethoprim; SMX: sulfamethoxazole; FODMAP: fermentable oligosaccharides, disaccharides, monosaccharides, and polyols.

## Data Availability

No new data were created or analyzed in this review.
